# Exploring the effects of serial and parallel elasticity on a hopping robot

**DOI:** 10.3389/fnbot.2022.919830

**Published:** 2022-08-24

**Authors:** Guoping Zhao, Omid Mohseni, Marc Murcia, Andre Seyfarth, Maziar A. Sharbafi

**Affiliations:** Lauflabor Locomotion Laboratory, Centre for Cognitive Science, Technical University of Darmstadt, Darmstadt, Germany

**Keywords:** serial elasticity, parallel elasticity, hopping robot, pneumatic artificial muscle, hybrid actuation

## Abstract

The interaction between the motor control and the morphological design of the human leg is critical for generating efficient and robust locomotion. In this paper, we focus on exploring the effects of the serial and parallel elasticity on hopping with a two-segmented robotic leg called electric-pneumatic actuation (EPA)-Hopper. EPA-Hopper uses a hybrid actuation system that combines electric motors and pneumatic artificial muscles (PAM). It provides direct access to adjust the physical compliance of the actuation system by tuning PAM pressures. We evaluate the role of the serial and parallel PAMs with different levels of compliance with respect to four criteria: efficiency, performance, stability, and robustness of hopping against perturbations. The results show that the serial PAM has a more pronounced impact than the parallel PAM on these criteria. Increasing the stiffness of the serial PAM decreases the leg stiffness of the unloading phase during hopping. The stiffer the leg, the more efficient and the less robust the movement. These findings can help us further understand the human hopping mechanism and support the design and control of legged robots and assistive devices.

## 1. Introduction

Stability, performance, robustness, and efficiency are four essential metrics to evaluate controlled machines, such as legged locomotor systems. Since legged biological systems such as humans are still far beyond robots in providing an optimal trade-off between these metrics, understanding the biomechanics of legged locomotion can help us develop robust and efficient high-performance robots (e.g., versatile gait). Interestingly, the principles of human locomotion can be explained and reproduced by simple template models (Full and Koditschek, 1999). For instance, it has been shown that the spring-loaded inverted pendulum (SLIP) model can generate human-like bouncing gaits (Blickhan, 1989; Geyer et al., 2006). The model behavior can be further tuned by introducing neuromuscular control (Geyer et al., 2003; Schumacher and Seyfarth, 2017; Davoodi et al., 2019). These underlying mechanics and motor control principles of human locomotion can help design efficient and robust legged robots (Hosoda et al., 2008; Zhao et al., 2020; Galljamov et al., 2021).

Besides the controller, the actuator properties are also important for generating efficient and robust locomotion. Actuation in biological systems consists of a redundant network of muscles, tendons, and ligaments, which result in inherent joint compliance (Farley et al., 1998). Bioinspired compliant actuators have been applied and tested on miscellaneous robots (Van Ham et al., 2009). They have shown many benefits in the context of legged locomotion, such as increasing energy efficiency, decreasing peak motor power, protecting motors from high force impacts, and reducing the number of sensors (Iida, 2005; Grimmer et al., 2014; Rouse et al., 2014). This elasticity in robotic systems can be induced either virtually by the control of rigid actuators or physically replicated by adding compliant elements to the robot structure (e.g., Zhao et al., 2020; Galljamov et al., 2021). Having a serial arrangement of compliance decouples the motor inertia from the load, which is favorable for shock absorbance and energy buffering (Grimmer et al., 2012). However, it cannot lower the peak torque requirements and is limited in terms of energy consumption reduction (Beckerle et al., 2016). In contrast, parallel elasticity can overcome these limitations as it can modify the system's natural dynamics by directly affecting the motor torque (Sharbafi et al., 2020; Mohseni et al., 2021). Therefore, a system with both series and parallel compliance can be very flexible for various locomotion tasks' torque and power requirements.

One way to implement such a bioinspired actuation system in robots is using variable impedance actuators (VIA) (Van Ham et al., 2007; Vanderborght et al., 2013; Wolf et al., 2015). In most VIA designs, the same type of actuator is used for actuation and impedance adjustment. In Ahmad Sharbafi et al. (2017), we introduced the electric-pneumatic actuation (EPA) as a hybrid VIA to benefit from the dynamical characteristics of different actuation technologies. The EPA design combines the precise and high-bandwidth characteristics of electric motors with the compliant properties of pneumatic artificial muscles (PAMs) (Mohseni et al., 2020). EPA adjusts the actuator impedance by changing the PAM pressure. The muscle-like properties of the PAM (Klute et al., 1999; Mohseni et al., 2020) provide robots with the required intrinsic passive behaviors for different locomotion tasks.

Another essential aspect of legged locomotion is the morphological design and the arrangement of different elements in the locomotor system. In this respect, compliance can be implemented in the joint with two different configurations: serial and parallel with the existing actuators. Different configurations have strong influences on both the requirements for the actuators (e.g., torque, power) and the locomotion behaviors (Van Ham et al., 2009; Yesilevskiy et al., 2015; Verstraten et al., 2016). For example, it has been shown that the optimal serial elastic arrangement is significantly more energetically efficient than the optimal parallel elastic arrangement for a hopper robot actuated with a geared electric DC motor (Yesilevskiy et al., 2015). Identifying the functionality of the serial and parallel elastic component in the joint can help us find the optimal design of the legged robot in the future. For instance, the robot can achieve better stability and efficiency while having smaller electric motors by adding an appropriate serial or parallel elastic element to the joint. With the EPA design, we can combine different serial and parallel arrangements and alternate between them (Sharbafi and Mohammadi Nejad Rashty, 2021). This enables us to find a potential unique arrangement of compliant elements that can optimize a multi-objective function which consists of the following four criteria: efficiency, performance, stability, and robustness.

Therefore, in this paper, we investigate the effects of serial and parallel compliance on hopping using the recently developed EPA-Hopper robot with a hybrid EPA actuator at the knee joint. EPA-Hopper is an extension of our previous EPA designs (Sharbafi et al., 2020; Zhao et al., 2020; Galljamov et al., 2021). It is a testbed that can be used to investigate different arrangements and effects of elasticity at the knee joint. We performed multiple perturbed hopping experiments with different PAM settings and evaluated the hopping behaviors with respect to the four criteria. We hypothesize that there is no unique compliance arrangement (a set of serial and parallel PAM pressure) that can optimize all four metrics. To better understand the role of motor control and joint compliance (i.e., serial and parallel PAMs), we implemented the bioinspired neuromuscular reflex-based hopping control where the motor was controlled as a virtual muscle tendon complex (Zhao et al., 2020). The interactions between the physical (i.e., the PAMs) and virtual compliance (i.e., the motor) and their effects on the leg level behaviors were analyzed in this study. In addition, to demonstrate the extendability of the findings, we performed a verification experiment with a more complex three-segmented legged robot EPA-Hopper II.

## 2. Methods

### 2.1. EPA-Hopper robot

**Figure 1:**
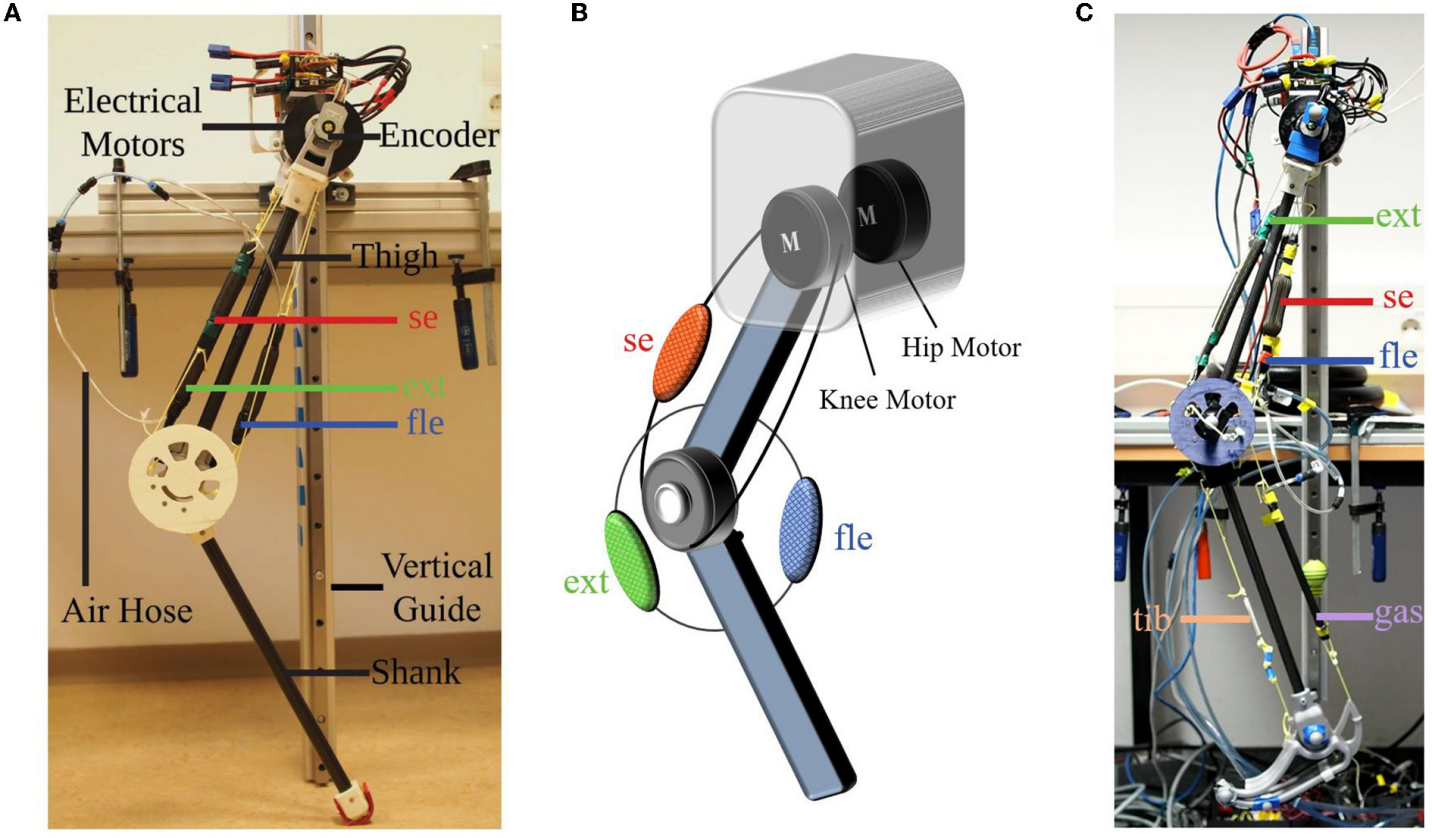
Electric-pneumatic actuation (EPA)-Hopper robot. **(A)** A photo of EPA-Hopper. **(B)** The schematic illustration of EPA-Hopper's actuation system. EPA-Hopper is a two-DoF leg co-actuated by two electric motors and three pneumatic artificial muscles (PAM) acting on the knee joint. **(C)** A photo of EPA-Hopper II, an extension of EPA-Hopper comprising an ankle joint and a foot. *se, fle*, and *ext* indicate the serial, flexor, and extensor PAMs, respectively. *gas* and *tib* indicate the biarticular gastrocnemius PAM and the ankle dorsiflexion spring, respectively.

The EPA-Hopper robot (shown in Figure 1A) was used for the experimental studies in this work. It is an extension of our previously developed MARCO Hopper II robot (Galljamov et al., 2021) and the GURO robot (Zhao et al., 2020). It is a two-segmented robotic leg consisting of hip and knee joints. The robot body is constrained by a vertical rail guide so that it can hop vertically. Both hip and knee joints are actuated separately by two brushless direct current motors (E8318-120KV, Hymotor, China). The robot's whole body center of mass (CoM) position is very close to the hip joint. For reducing the leg moment of inertia, both motors are placed co-axially on the hip, as shown in Figure 1. In this setup, the hip is direct driven while the knee uses a rope and pulley mechanism with a ratio of 5:1. The electronics used for EPA-Hopper are the same as the GURO robot. Please find the detailed description in Zhao et al. (2020).

In addition to the two electric motors, the robot is equipped with three PAMs. One PAM is in serial with the knee motor (serial, length 12cm). Two PAMs are in parallel with the knee joint for extending (extensor, length 22cm) and flexing (flexor, length 16cm) the knee joint (Figures 1A,B), respectively. The length and lever arm of the PAMs are selected based on the knee joint range of movement during hopping and our pilot PAM testing experiments (Mohseni et al., 2020). Each PAM is equipped with two continuous valves (PVQ-31, SMC, Japan) and a pressure sensor (PSE530, SMC, Japan) for controlling the PAM pressure. The physical properties of the robot are summarized in Table 1.

**Table 1:** Electric-pneumatic actuation (EPA)-Hopper physical properties.

**Parameters**	**Value [unit]**
Shank length *l*_*s*_	0.46m
Thigh length *l*_*t*_	0.42m
Driver pulley diameter *r*_1_	0.02m
Follower pulley diameter *r*_2_	0.10m
Shank mass *m*_*s*_	0.37kg
Thigh mass *m*_*t*_	1.56kg
Hip mass *m*_*h*_	1.24kg
Motor maximum torque τ_*m*_	4Nm
Motor maximum speed ω_*m*_	2,600 rpm

For verification of the results, an extension of the EPA-Hopper comprising a third segment (i.e., an ankle joint with a foot) was utilized (shown in Figure 1C). This robot, named EPA-Hopper II, employs an additional biarticular PAM which crosses the knee joint and the ankle joint (i.e., it represents the gastrocnemius muscle in the human leg). It also uses a spring, which imitates the tibialis muscle for the ankle dorsiflexion. A detailed description of the robot can be found in our previous study (Mohseni et al., 2022).

### 2.2. Reflex-based control

It has been shown that the bio-inspired neuromuscular reflex control with positive force feedback (PFF) can produce human-like stable and robust hopping behavior both in simulation (Geyer et al., 2003; Schumacher and Seyfarth, 2017) and in the robot (Zhao et al., 2020). Therefore, we implemented the same control scheme as in our previous study (Zhao et al., 2020) on the EPA-Hopper. During the stance phase, the knee motor was controlled as a virtual Hill-type muscle tendon complex (MTC) in which the muscle stimulation is proportional to the MTC force. The virtual MTC force *F*_*mtc*_ can be described as a function of the muscle activation signal *A*, muscle length *l*_*m*_, muscle velocity *v*_*m*_, MTC length *l*_*mtc*_, and MTC velocity *v*_*mtc*_.


(1) $$\begin{array}{r} F_{mtc}=f\left(A,l_{m},v_{m},l_{mtc},v_{mtc}\right) \end{array}$$

The relation between the muscle activation *A*(*t*) and the PFF reflex are defined by the following equations


(2) $$\begin{array}{r} \tau \frac{\text{d}A\left(t\right)}{\text{d}t}=S\left(t\right)-A\left(t\right) \end{array}$$

(3) $$\begin{array}{c} S(t)=\left\{ \begin{array}{l} S_{0}\;t\leq \delta t\\ \;S_{0}+G_{F}F_{mtc}(t-\delta t)\;t>\delta t \end{array}\right. \end{array}$$

where τ is a time constant, *S*(*t*) is the muscle stimulation signal, *S*_0_ is the constant stimulation bias, δ*t* is the time delay in the feedback pathways, and *G*_*F*_ is the force feedback gain. A detailed description of the virtual MTC model and the PFF can be found in our previous study (Zhao et al., 2020).

*G*_*F*_ was manually tuned so that the robot can generate stable hopping if the air pressure in each PAM is not lower than 0.4MPa. The hip motor torque was set to 0 during the stance phase. During the flight phase, both hip and knee motors were position controlled. Please check (Zhao et al., 2020) for a detailed description of the controller. The controller was implemented with Matlab Simulink xPC (Matlab R2019a, Mathworks, USA) in real-time at 1kHz.

### 2.3. Robot perturbation experiment

We conducted a perturbed hopping experiment with the EPA-Hopper robot in 27 different conditions (combinations of PAM pressures). Each PAM pressure was tuned to a predefined constant value before the hopping experiments. The same control parameters of the reflex-based controller were used in all experiments. Based on previous studies (Hosoda et al., 2010; Ahmad Sharbafi et al., 2017; Galljamov et al., 2021), in order to have a detectable change in the PAM elasticity and the robot hopping behavior, three different PAM pressures (i.e., 0.4, 0.5, and 0.6MPa) were used in this work. We chose 0.4MPa as the minimum pressure because the robot cannot generate stable hopping with lower pressure values in the PAMs. Then, all valves were closed during the experiment. In the experiments, the PAM pressure changes are less than 3, 10, and 5% for the serial, extensor, and flexor PAMs, respectively. Before each experiment, we adjusted each PAM's rope length so that each PAM was pretensioned when the knee joint angle reaches the predefined flight phase joint angle. EPA-Hopper started hopping after it was dropped on a wooden block from 10cm height. To introduce the ground dropping perturbation during hopping, the wooden block (thickness 5cm) was removed during the flight phase after 30 hops. The experiment was terminated after another 30 hops. Ground reaction forces were measured by a force plate (Kistler, Germany) at 1kHz. A 3D motion capture system (Qualisys, Sweden) recorded the robot's hip and foot position at 500Hz.

### 2.4. Hopping behavior evaluation metrics

The hopping behavior is evaluated with four criteria: efficiency, performance, stability, and robustness.

#### 2.4.1. Efficiency

Hopping efficiency can be quantified by the normalized energy consumption of the robot (*E*/*h*), which is defined as the ratio between the robot's energy consumption (*E*) during a hop cycle and the robot's stable hopping height (*h*). The PAMs do not consume energy during hopping because all valves are closed. Therefore, we only consider the electric energy consumption of the hip and knee motors because the energy consumption of the other parts (e.g., sensors, control boards, etc.) is constant with respect to different hopping behaviors. The energy consumption *E* is calculated as


(4) $$\begin{array}{r} E=\overset{n}{\underset{i=1}{\sum }}\left(V_{i}^{\text{hip}}I_{i}^{\text{hip}}+V_{i}^{\text{knee}}I_{i}^{\text{knee}}\right)\Delta t \end{array}$$

where *V* and *I* denote the motor voltage and current measured by the motor driver. *i* = 1 and *i* = *n* denote the first and the last time instance of a hopping cycle, respectively. Δ*t* is 1ms because the data are recorded at 1kHz. The hopping height *h* is defined in Section 2.4.2.

#### 2.4.2. Performance

Hopping performance is quantified by the hopping height (*h*), which is computed as follows


(5) $$\begin{array}{r} h=h_{max}-h_{td} \end{array}$$

where *h*_*max*_ and *h*_*td*_ denote the maximum hip height (apex) during the flight phase and the hip height at the touch-down moment, respectively. The touch-down moment is defined as the timing when the vertical ground reaction force is larger than 5N. The mean hopping height of the 15 hops before the perturbed hop is considered the stable hopping height.

#### 2.4.3. Stability

A small perturbation can be used to verify the stability of a cyclic motion. The robot hopping motion can generate a stable limit cycle if the robot states return to the same equilibrium after the perturbation. In theory, if the resulting limit cycle after the perturbation is different from the original one, the original equilibrium is not stable. However, with uncertainties in the hardware setup, a slight deviation from the original cycle is inevitable. Thus, to verify stability in the robot experiment, we define a small range around the cycle. To simplify this procedure, we reduce the system order using the Poincaré return map. We utilize the apex height to define the Poincaré section and the difference between hopping height before and after perturbation (Δ*h*) to quantify hopping stability, i.e.,


(6) $$\begin{array}{r} \Delta h=|h_{\text{before}}-h_{\text{after}}| \end{array}$$

where *h*_before_ and *h*_after_ denote the stable hopping height before and after the perturbation, respectively. By defining a threshold (e.g., 10% of *h*_before_), the robot responses can be divided into stable and unstable solutions.

#### 2.4.4. Robustness

In control theory, robustness is evaluated by identifying the maximum range of perturbations or uncertainties that cannot destabilize the stable response of the system (e.g., gain and phase margin) (Zhou and Doyle, 1998). Such a measurement is challenging in robotic experimental setups such as our EPA-Hopper. Linear control system theory also shows that the system with a faster settling time (closer poles to the origin in discrete systems represented by Poincaré map) can have higher robustness against parameter variations (e.g., higher gain margin). Therefore, the recovery time from the perturbation can be used as an indicator for robustness evaluation. It has also been used in biomechanical studies to evaluate robustness against perturbation (Zelei et al., 2021). Here, we measure the number of hops (*N*) required to return to the 5% neighborhood of the stable hopping height after convergence. This measure is used to evaluate the robustness of hopping.

## 3. Results

Here, we explain the findings of the perturbation experiment and analyze the effects of PAMs in terms of energy consumption, hopping height, stability, and robustness. In addition, the correlation of the leg stiffness, the PAM pressure, and the four evaluation metrics are presented. This provides a new perspective for further understanding the effects of compliance on dynamic locomotion.

### 3.1. PAM pressure vs. evaluation metrics

#### 3.1.1. Normalized hopping energy consumption

**Figure 2:**
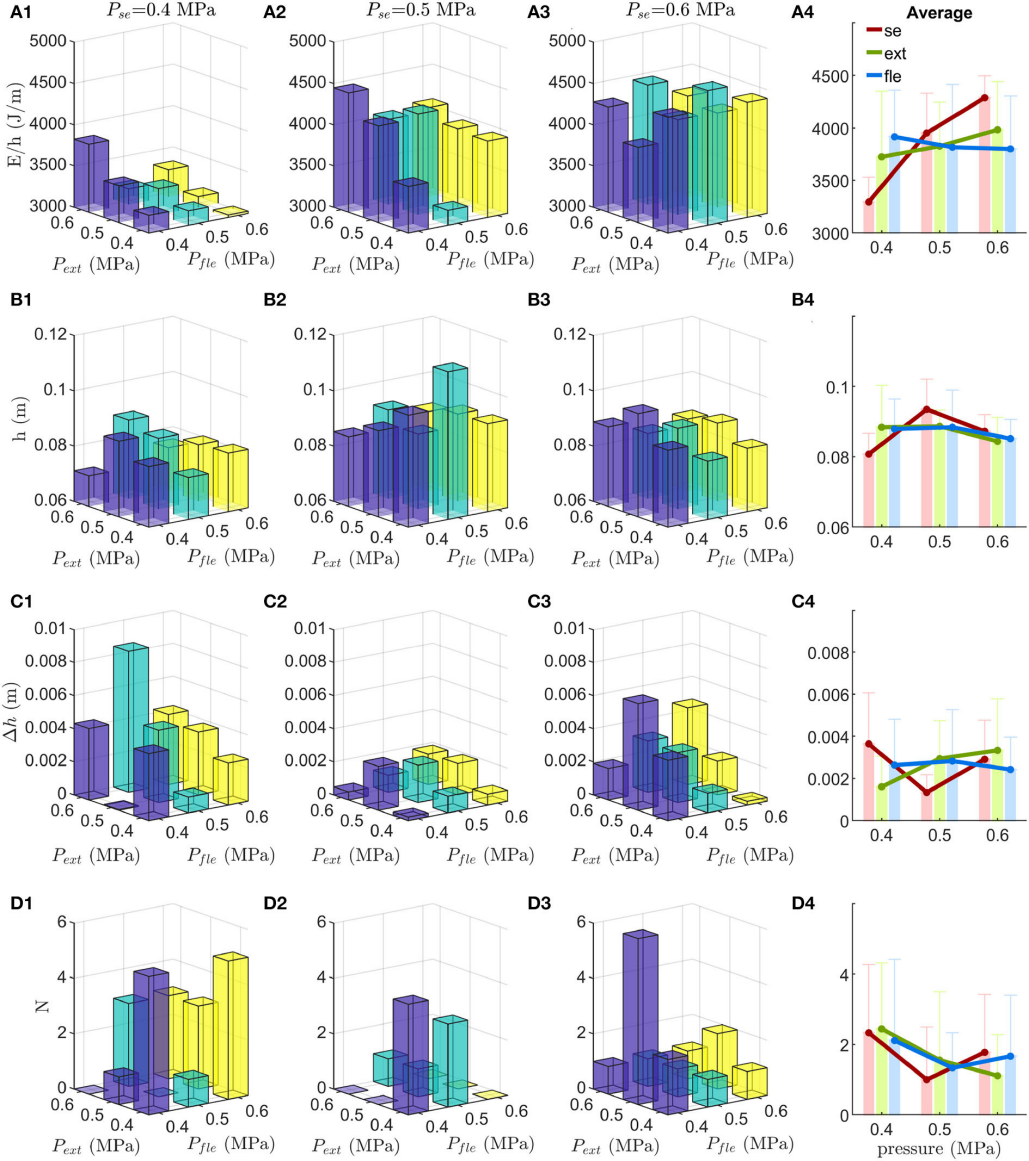
Experiment results on EPA-Hopper. The four hopping metrics vs. different PAM pressure (*P*). The subscripts *se, fle*, and *ext* indicate the serial, flexor, and extensor PAMs, respectively. **(A)** Normalized energy consumption (*E*/*h*). **(B)** EPA-Hopper stable hopping height (*h*). **(C)** Stable hopping height difference (Δ*h*) as a measure of stability. **(D)** Hopping robustness in terms of the number of hops (*N*) required to recover from the perturbation. The figures in the 4th column show the average and standard deviation of *E*/*h*, *h*, Δ*h*, and *N* with respect to different pressures in serial, flexor, and extensor PAMs.

The mean normalized hopping energy consumption of the 15 hops before perturbation is shown in Figures 2A1–A3. The results show that, in general, low pressure in the serial PAM results in lower normalized energy consumption. The lowest normalized energy consumption is 3,028 J/m (*P*_*se*_ = 0.4MPa, *P*_*fle*_ = 0.6MPa, *P*_*ext*_ = 0.4MPa). Interestingly, the normalized energy consumption of the highest hopping height which occurs at PAM pressure settings of (*P*_*se*_ = 0.5MPa, *P*_*fle*_ = 0.5MPa, *P*_*ext*_ = 0.4MPa) is relatively low (3179J/m), as shown in Figure 2A. On average, the energy consumption increases with increasing pressure in the serial PAM (Figure 2A4).

#### 3.1.2. Hopping height

The mean hopping height of the 15 hops before perturbation is considered the hopping height (shown in Figures 2B1–B3). EPA-Hopper can achieve stable hopping with all PAM pressure settings. The highest hopping height is 11.3 cm (*P*_*se*_ = 0.5MPa, *P*_*fle*_ = 0.5MPa, *P*_*ext*_ = 0.4MPa). The lowest hopping height is 7.1cm (*P*_*se*_ = 0.4MPa, *P*_*fle*_ = 0.4MPa, *P*_*ext*_ = 0.6MPa). On average (Figure 2B4), the hopping height with medium pressure in the serial PAM (*P*_*se*_ = 0.5MPa, *h* = 9.4±0.8cm) is higher than the other two pressure settings (*P*_*se*_ = 0.4MPa, *h* = 8.1±0.6cm; *P*_*se*_ = 0.6MPa, and *h* = 8.7±0.5cm). The average hopping height does not change consistently with respect to different pressures in the flexor and extensor PAM (Figure 2B4).

#### 3.1.3. Hopping stability

EPA-Hopper can regain stable hopping settings after the 5 cm ground dropping perturbation for all PAM pressure combinations. The hopping height difference Δ*h* (defined in 6) is lower than 10% of the original hopping height in most of the PAM pressure settings (25 out of 27), as shown in Figures 2C1–C3. These results show that the combination of different PAM pressures with a matching reflex-based controller enables the EPA-Hopper to recover from perturbation and reach the previous hopping height.

Medium pressure in the serial PAM results in both the lowest average hopping height difference and the most stable hopping (i.e., the lowest hopping height difference variation) (*P*_*se*_ = 0.4MPa, Δ*h* = 0.36 ± 0.24cm; *P*_*se*_ = 0.5MPa, Δ*h* = 0.13 ± 0.08cm; *P*_*se*_ = 0.6MPa, Δ*h* = 0.29 ± 0.19cm, Figure 2C4).

#### 3.1.4. Hopping robustness

The required number of hops (*N*) to recover from perturbation is used to evaluate the hopping robustness. Figures 2D1–D3 shows that the robot could immediately recover or needs a few hops (maximum 6 hops) to converge to stable hopping after 5cm ground dropping perturbation. The medium pressure in the serial PAM yields the most robust hopping behavior (*P*_*se*_ = 0.5MPa, average *N* = 1.00; *P*_*se*_ = 0.6MPa, average *N* = 1.78; *P*_*se*_ = 0.4MPa, average *N* = 2.33) (Figure 2D4). It requires more hops (4–6 hops) to recover from perturbations if the pressure in the serial PAM is low and the pressure in the flexor PAM is high. On the contrary, EPA-Hopper can reach stable hopping right after perturbation if the pressure in the serial PAM is medium and the pressure in the flexor PAM is high. The average *N* decreases (i.e., hopping robustness improves) with increasing extensor PAM pressure (Figure 2D4). This trend is consistent when comparing the *N* of the individual trial (shown in Figures 2D1–D3). The higher extensor pressure shows higher robustness in all cases except three.

### 3.2. Leg stiffness vs. serial PAM pressure and the metrics

**Figure 3:**
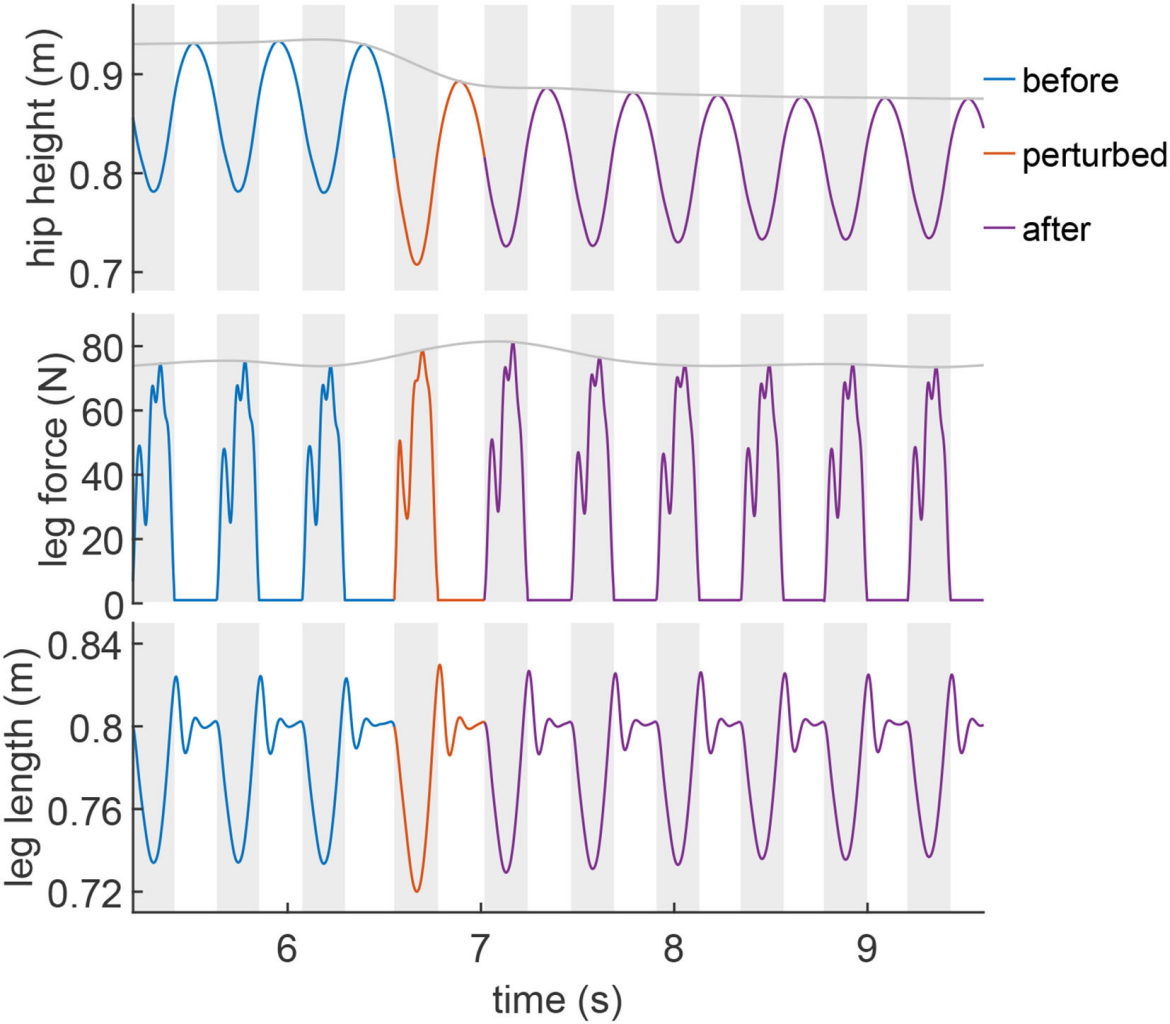
Example of the measured hip height, leg force, and leg length of EPA-Hopper during a perturbed hopping experiment (*P*_*se*_ = 0.4MPa, *P*_*ext*_ = 0.5MPa, *P*_*fle*_ = 0.6MPa). The perturbed hop is shown in orange. The blue and velvet colors denote the hops before and after the perturbed hop. Gray shaded areas denote the stance phases.

**Figure 4:**
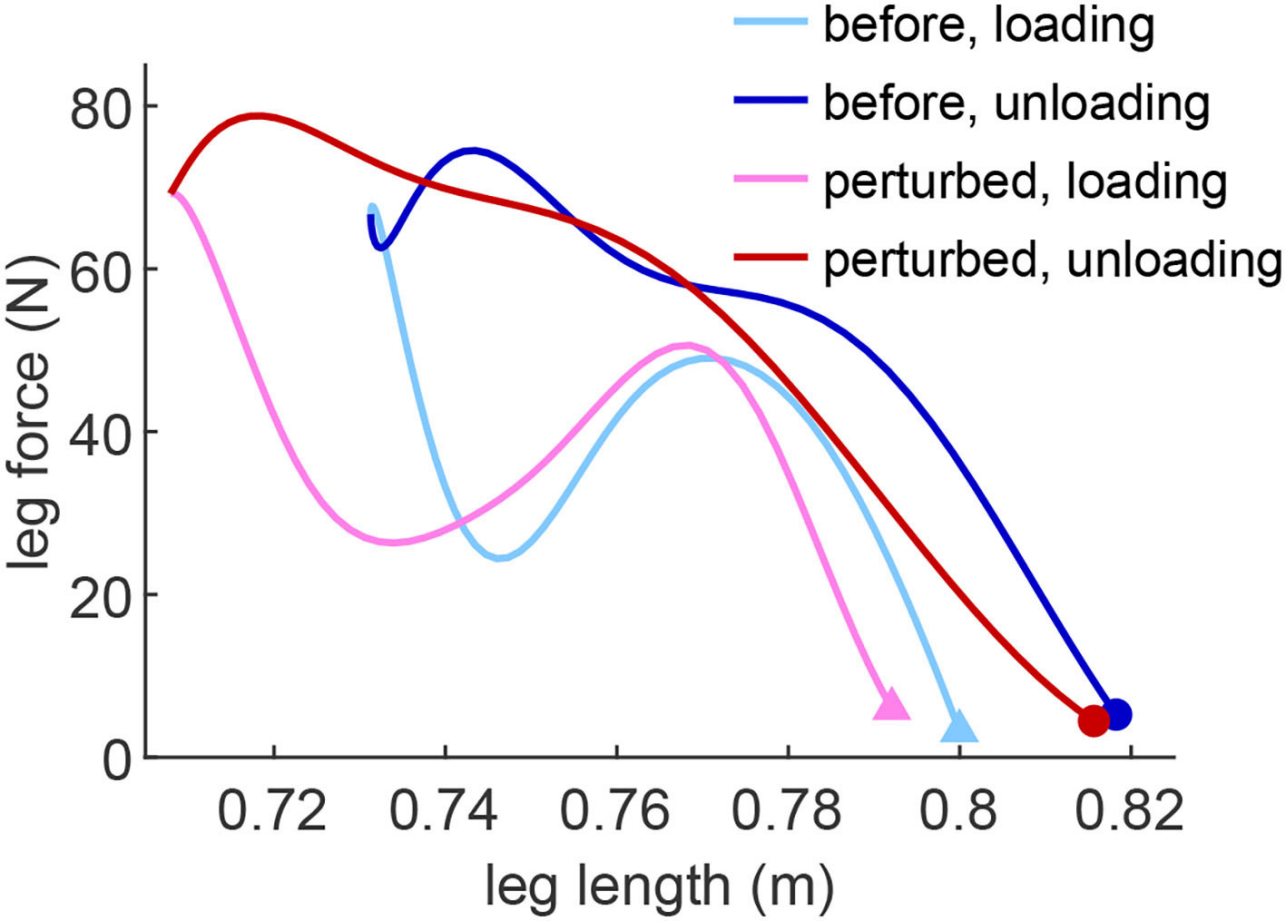
Example of the leg force-length curve of the perturbed hop (in red) and the stable hop (in blue) before the perturbation (*P*_*se*_ = 0.4MPa, *P*_*ext*_ = 0.5MPa, *P*_*fle*_ = 0.6MPa). The leg force-length curve is divided into the loading phase (the CoM moving downwards during the stance phase) and the unloading phase (the CoM moving upwards during the stance phase). The triangle and the dot markers denote the touch-down and take-off, respectively.

The spring-like leg behavior can be observed in human hopping experiments (Blickhan, 1989). In order to investigate how EPA-Hopper leg compliance changes with different PAM pressures, the leg stiffness during the loading (CoM moving downwards) and unloading (CoM moving upwards) phases are computed and analyzed. Figure 3 shows a typical leg response (hip height, leg force, and leg length) during a perturbed hopping experiment. The hopping behavior is quite stable both before and after the perturbation. In both perturbed and unperturbed hops, the leg force-length relation during the unloading phase is more linear (spring-like) than during the loading phase (Figure 4). The leg stiffness is calculated by linear fitting the force-length curve.

**Figure 5:**
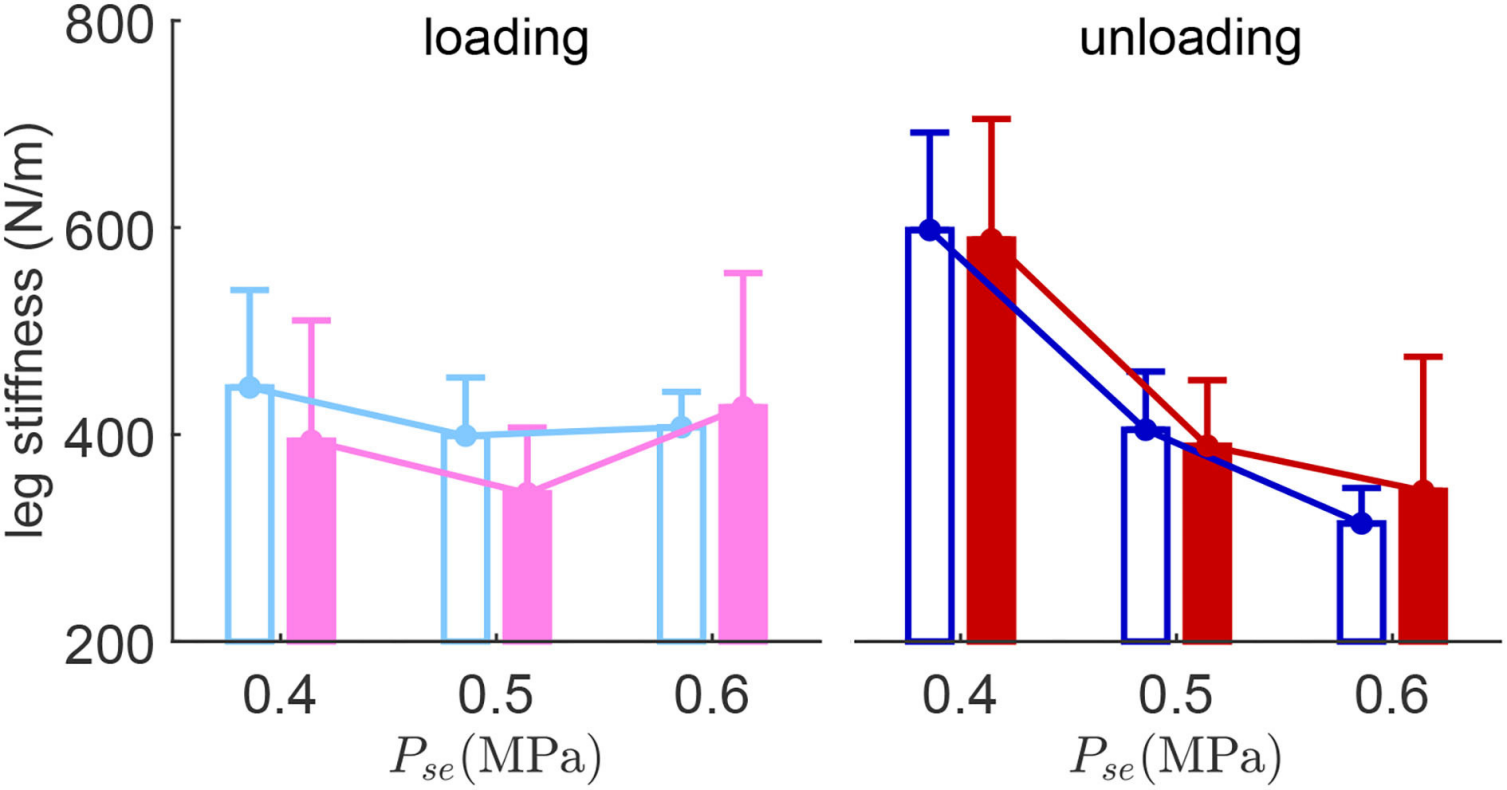
Leg stiffness during the loading phase (the stance phase when the CoM moving downwards) and the unloading phase (the stance phase when the CoM moving upwards). Error bars denote the SD. Solid and hollow bars denote the perturbed hop and the unperturbed hop (hops before the perturbation) conditions, respectively.

The results show that the average leg loading stiffness does not change too much with respect to different serial PAM pressures (Figure 5). On the contrary, the average leg unloading stiffness has a clear decreasing trend with increasing serial PAM pressure in both perturbed and unperturbed hops. Although this observation might seem surprising, it could be explained by the biological motor control principles (see Section 4.2.1). The average leg unloading stiffness of the unperturbed hopping is similar to the perturbed hop. In the low and medium serial PAM pressure settings, the average leg loading stiffness of the perturbed hop is lower (*P*_*se*_ = 0.4MPa, 11.6%; *P*_*se*_ = 0.5MPa, 13.9%) than the hops before the perturbation.

**Figure 6:**
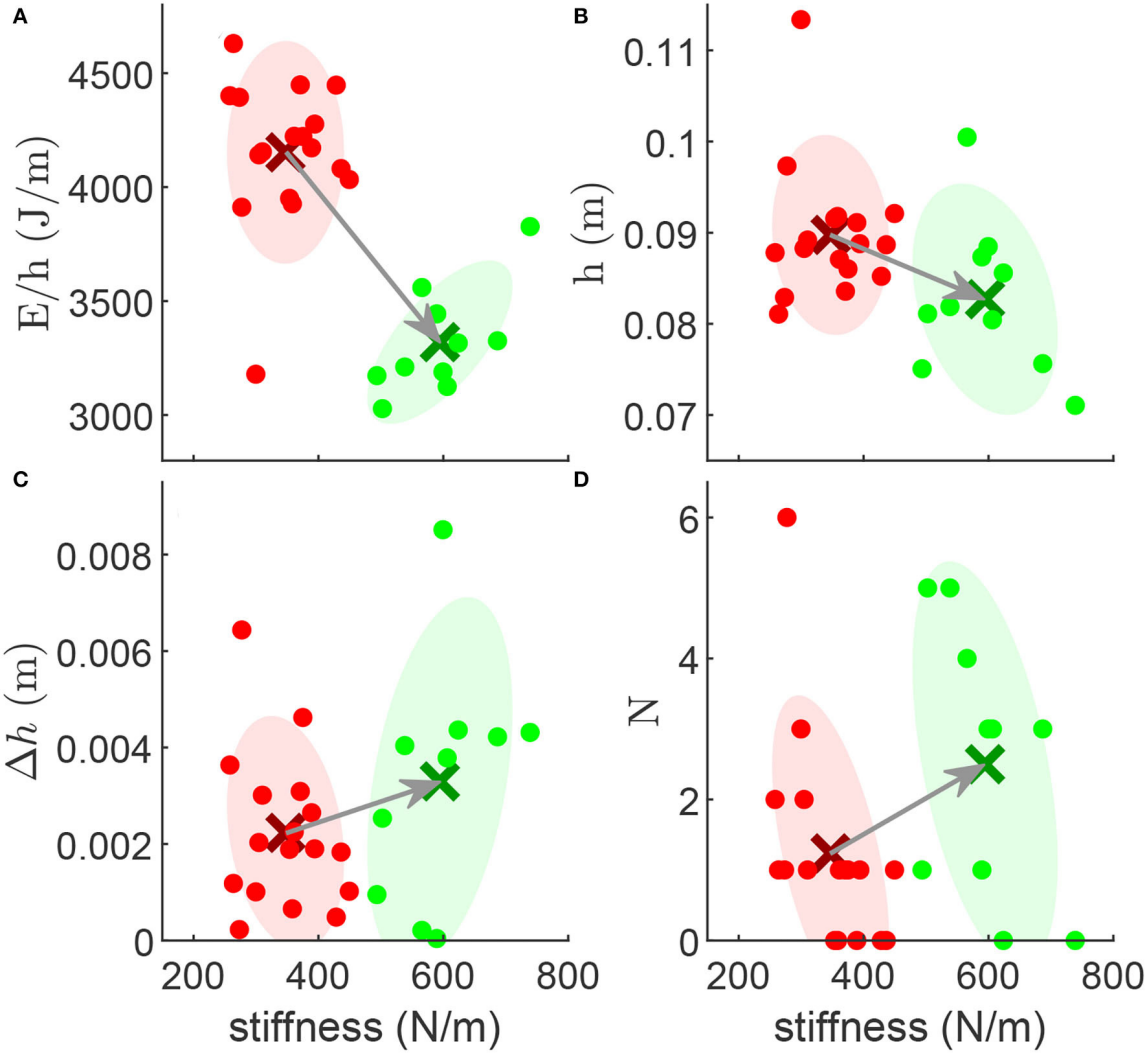
The relation between the leg unloading stiffness and the **(A)** normalized energy consumption (*E*/*h*), **(B)** hopping height (*h*), **(C)** stable hopping height difference (Δ*h*), **(D)** number of hops (*N*) required to recover from the perturbation. The red and green dots denote the low stiffness and high stiffness conditions, respectively. The red and green ellipses denote the covariance of the low and high stiffness data groups (with 1 SD), respectively. The cross markers denote the mean of the data groups.

There is no clear correlation between the leg loading stiffness and the evaluation metrics. The relation between the leg unloading stiffness and the hopping energy consumption, height, stability, and robustness is shown in Figure 6. Considering 450N/m as a borderline, we cluster the leg stiffness values to the high stiffness group and the low stiffness group. The energy consumption and the hopping height of the low stiffness group are, respectively, 25.1% and 8.5% higher than those of the high stiffness group (Figures 6A,B). The stable hopping height difference Δ*h* and the number of recovery-hops *N* of the low stiffness group are 32.3% and 50.1% lower than the high stiffness group (Figures 6C,D), respectively.

### 3.3. Hopping with EPA-Hopper II

**Figure 7:**
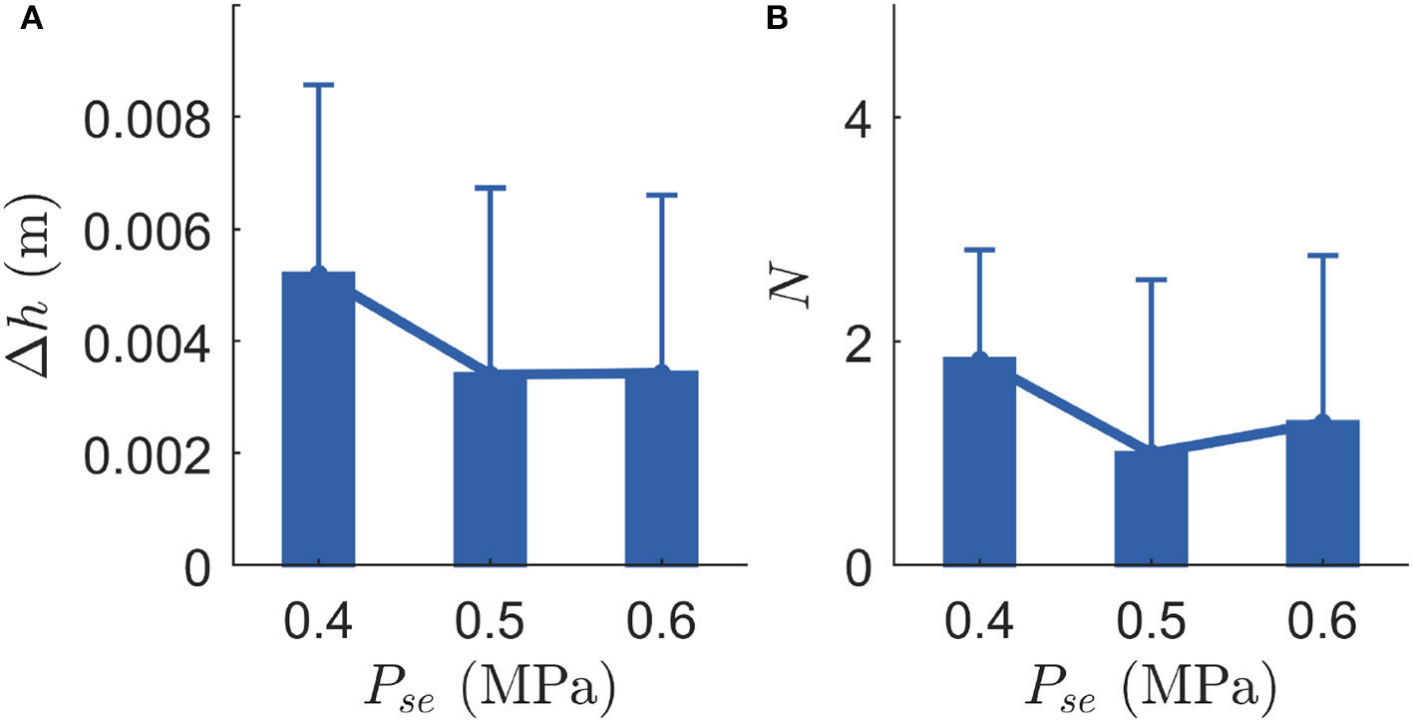
Experiment results on EPA-Hopper II. Each experimental condition was repeated six times. *P*_*se*_ indicates the serial PAM pressure. Error bars indicate one SD. **(A)** Stable hopping height difference (Δ*h*, hopping stability). **(B)** The number of hops required to recover from the perturbation (*N*, hopping robustness).

Stability and robustness are two key criteria for evaluating the robot in perturbation scenarios. In order to show the repeatability of the perturbation responses and support the findings, we performed a new experiment with a more complex robot EPA-Hopper II which has a three-segmented leg (including thigh, shank, and foot, Figure 1). In this new experiment, we considered the same PAM configurations for the knee joint, while a biarticular PAM (i.e., gastrocnemius) was used for the ankle joint. To support the findings of the role of the serial PAM, we kept the parallel PAMs' pressures to the medium value (i.e., *P*_*ext*_ = 0.5MPa, *P*_*fle*_ = 0.5MPa) and performed perturbed hopping experiments with different serial PAM pressures (i.e., 0.4MPa, 0.5MPa, and 0.6MPa). In order to minimize the gastrocnemius PAM's potential influences on the hopping behavior and ensure a reasonable ankle joint motion, the gastrocnemius PAM pressure was set to 0.1MPa. To prevent ankle hyper-extension in the flight phase, we added a tibialis spring stiffness and its rest length was selected based on a previous study (Mohseni et al., 2022). The motor control parameters and the parallel PAM rest lengths were the same as in the previous EPA-Hopper experiment. Each condition was repeated six times. The same perturbation (5 cm ground drop) was introduced during hopping. The results (Figure 7) show that, although the robot is more complex (with an additional ankle joint), the serial PAM has very similar influences as the simpler robot (Figures 2C4,D4) on the hopping stability and hopping robustness. These results support our previous findings and demonstrate the extendability of the findings to a more complex system.

## 4. Discussion

In this work, we investigated the effects of different PAM pressure settings (serial, extensor, and flexor) on the hopping behavior with four evaluation metrics: efficiency, performance, stability, and robustness. We found that, compared to the parallel PAMs (i.e., extensor and flexor), the serial PAM has a more pronounced impact on the hopping behavior. Interestingly, the results show that increasing serial PAM stiffness results in lower leg stiffness during the unloading phase. The leg stiffness results also indicate a trade-off between energy efficiency and robustness. These findings can help us further understand the role of elastic components in dynamic locomotion and develop more efficient and robust robots and assistive devices.

With a neuromuscular controller using PFF (Geyer et al., 2003; Zhao et al., 2020), the motor represents the muscle, while the passive series and parallel compliance in the biological actuation system (e.g., tendon and ligament) are represented by the PAMs. Interestingly, this combination of passive and active compliance can generate sufficient joint torque to recover from perturbation with a deadbeat response (see Figure 3) without changing the control parameters. A unique feature of this study that cannot be easily performed in human experiments is the ability to change the passive element properties (i.e., modulate the stiffness of series and parallel compliance) to understand their roles in generating a hopping movement. We can evaluate the hopping behavior in different conditions with the four metrics mentioned earlier. The interaction between the virtual compliance introduced by the reflex-based control of the electric motor and the physical compliance (adjusted by PAM pressure) and its impact on hopping behavior is scrutinized.

In the following, first, we discuss the effects of different compliant elements (PAMs) on movement behavior. Then, the relation between the physical compliance at the knee joint and the leg stiffness is analyzed. Finally, the influence of leg stiffness on hopping behavior is elaborated.

### 4.1. Serial compliant tendon facilitates hopping control

Among the three PAMs added in parallel and series to the knee joint, the serial PAM demonstrated the most consistent influence on different movement criteria. In addition, the results also support our hypothesis that there is no unique set of PAM pressures that can optimize all metrics simultaneously. In the following, we discuss the role of different PAMs in producing different hopping behaviors.

#### 4.1.1. Softening tendon could improve energy efficiency

Our results show that increasing the serial PAM pressure reduces energy efficiency (see Figure 2A4). It has been shown that increasing the ground stiffness can result in increasing leg work (consequently energy consumption) during hopping (Moritz and Farley, 2005). The ground compliance can be considered serial compliance to the leg for vertical hopping. Therefore, the findings from Moritz and Farley (2005) support our results that stiffening the serial elastic element (compliant ground) can result in increased energy consumption for hopping. In addition, a study of human hopping analyzes at different frequencies using a serial elastic actuator model at different joints also showed similar relation between energy consumption and serial spring stiffness (Rashty et al., 2021).

#### 4.1.2. Robustness improvement with serial tendon adjustment

Medium pressure in the serial PAM provides the most stable, robust, and highest performance (Figures 2B4,C4,D4). The improved stability and robustness against perturbations mean that the serial elasticity, which replicates tendon behavior in muscle-tendon-complex (MTC), could facilitate shock absorption during perturbed hopping. These findings are in line with human hopping experimental studies (Dick et al., 2021). In similar perturbed hopping experiments with humans, Dick et al. (2021) showed that a ground drop introduces a phase shift in muscle activation which increases the MTC length and muscle forces. Consequently, the extra energy resulting from larger dropping will be dissipated easily within the intrinsic dynamics and corresponding motor control (Dick et al., 2021). The results of our experiments can be described with a similar neuromechanical control concept. The interaction between the muscle (electric motor) and the tendon (serial PAM) results in softening the leg during the loading phase of the perturbed hop if the tendon is not too stiff (see Figure 5). The force-velocity relation in the muscle model and the positive force feedback could generate the right timing if the tendon stiffness is appropriately tuned. With low and moderate tendon stiffness values, the automatic leg softening in the loading phase could dissipate the increased energy injected from perturbation which is missing when the tendon stiffness is high. This explains the highest robustness and stability with the moderate tendon stiffness (equivalently, the softest leg in the loading phase) as shown in Figures 2C,D.

#### 4.1.3. Parallel compliance effects

Depending on the serial PAM stiffness, the extensor and flexor PAMs can further influence efficiency, performance, and robustness. In general, if the parallel passive element is not tuned precisely, it could partly oppose the actuation and increase energy consumption. Hence, in addition to the stiffness of the parallel PAM, the parallel PAM's rest length, which determines the timing of its contribution, is also essential. This might be the reason that we did not find a clear trend in changing the parallel PAMs pressure on energy efficiency. Nevertheless, we found a pronounced influence of parallel PAM extensor on the robustness. Our results confirm that increasing extensor stiffness could improve hopping robustness. Improved damping of the impacts resulting from perturbation with increased extensor PAM stiffness could reduce the perturbation recovery time. This finding supports previous theoretical (Sharbafi et al., 2019) and experimental (Grabowski and Herr, 2009) studies on the advantages of parallel compliance.

### 4.2. Leg elastic property and hopping

Hitherto, we have explained the relation between active and passive compliance and its effects on hopping quality. Here, we first focus on the relation between the serial PAM pressure and the leg stiffness. Then, we analyze the leg elastic behavior and the consequences in the four evaluation metrics of hopping. As shown in Figure 4, the force-length behavior in the first half of the stance phase (loading) differs from the second half (unloading). This separation was also utilized in human hopping experimental analyzes (Moritz and Farley, 2005). The unloading phase shows a spring-like, almost linear force-length relation, while the loading phase reveals nonlinear behavior. These patterns are also consistent in the perturbed hop.

#### 4.2.1. Reciprocal relation of tendon and leg stiffness

Another surprising finding of this paper is the inverse relation between tendon stiffness and leg stiffness in the unloading phase. This phenomenon could be described by the paradoxical muscle movement introduced by Loram et al. (2004). In a standing experiment, it has been shown that the length of ankle muscles (soleus and gastrocnemius) increases while the MTC shortens during the backward swaying of the body. The reciprocal relation between the tendon and MTC stiffness can be seen in the similar relation between the serial PAM and the leg stiffness. In the robot unloading phase, the shortening of the virtual MTC length coincides with muscle lengthening if the tendon (i.e., serial PAM) stiffness is not sufficient. With the compensation of the virtual MTC stiffness generated by the electric motor, the resulting leg stiffness is sufficient to generate adequate energy for the next hop.

This behavior can also be explained by the underlying principles of our reflex control described in Section 2.2. For example, the nonlinear muscle force-length relationship and force-velocity relationship result in a muscle length and velocity dependent muscle stiffness during hopping. In addition, muscle activation can also modulate muscle stiffness. Therefore, the reflex control can produce reciprocal relation between the serial PAM and the MTC stiffness (generated by the electric motor). In other words, the overall knee joint stiffness can increase while the serial PAM stiffness decreases. As the leg stiffness is proportional to the knee joint stiffness in the robot, reducing the serial PAM pressure could increase leg stiffness as observed in our experimental results (Figure 5). In the hopping on compliant surface experiments, the same relation between the surface and leg stiffness can also be observed (Moritz and Farley, 2005).

#### 4.2.2. Phase-dependent leg stiffness adjustment improves hopping quality

Before discussing the different leg stiffness in loading and unloading phases, we want to interpret the robot hopping behaviors from the conceptual modeling perspective (Blickhan, 1989; Full and Koditschek, 1999). The SLIP model is the simplest (conservative) model which can explain bouncing gaits such as hopping and running (Blickhan, 1989; Geyer et al., 2006). In such a model, the body dynamics is simplified to a point mass on top of a massless spring. This combination of specific mass and spring stiffness results in a specific natural frequency of the system. In our experiments, the robot hopping frequency is between 2.0 and 2.4 Hz. This requires a leg stiffness ranging from 500 to 720 N/m (the mass of the robot is 3.2 kg). The results (Figure 6) show that increasing the leg stiffness to 600 N/m results in more efficient hopping. This means that if the parallel and serial PAMs can generate the leg stiffness in this range, the hopping frequency will be close to the natural frequency of the system which yields more efficient hopping. Therefore, by changing the mechanical compliance of the leg we could tune the natural frequency and achieve more efficient motion. This could be extended for other locomotion tasks (e.g., hopping at other frequencies).

For low and medium serial PAM pressure, the loading phase leg stiffness decreases in the perturbed hop while the unloading phase leg stiffness does not change in the perturbed hop (Figure 5). Comparison between the loading and unloading phases in the same experiments exhibits different patterns depending on the serial PAM pressure. With low, medium, and high stiffness in the serial PAM, the leg stiffness in the unloading phase is, respectively, higher, equal, and lower than that of the loading phase. This variation can be described by the amount of energy dissipated or injected in switching between the phases. Further analyzes in more extensive experiments are required to understand the relation between energy efficiency and tendon stiffness using the resulting leg force-length behavior. Interestingly, the leg stiffness turns negative shortly after landing during the loading phase (Figure 4). This is beneficial because it helps to dampen the landing impact energy and reduce the impact force.

The evolution of the four metrics based on leg stiffness unveils new aspects of hopping control. We did not find a clear pattern in the effects of loading stiffness on the four criteria. For this, we focused on the leg unloading stiffness. The results (Figure 6) show that increasing leg stiffness can reduce normalized energy [in line with findings in human hopping (Hobara et al., 2010)] while decreasing hopping height, stability, and robustness. We cannot find a unique strategy to change the leg stiffness which can improve all metrics. Therefore, we need to compromise between energy efficiency and the other metrics to find an appropriate hopping behavior according to the target condition.

In this study, we investigated the functionality of serial and parallel elasticity on hopping behaviors from four different criteria. We demonstrated the extendability of our key findings by an additional experiment with a more complex three-segmented robotic leg EPA-Hopper II. In future studies, the role of serial and parallel elasticity identified in hopping could be compared to other dynamic locomotion (e.g., running and jumping) with more complex robotic systems.

## Data availability statement

The raw data supporting the conclusions of this article will be made available by the authors, without undue reservation.

## Author contributions

GZ, MS, and AS were responsible for the conceptualization of this work. GZ, OM, and MM conducted the experiments. GZ analyzed the data. GZ, MS, and OM drafted the manuscript. All authors approved the final version of the manuscript.

## Funding

This work was supported by the German Research Foundation (DFG) grants number AH307/2-1, AH307/4-1, and SE1042/29-1.

## Conflict of interest

The authors declare that the research was conducted in the absence of any commercial or financial relationships that could be construed as a potential conflict of interest.

## Publisher's note

All claims expressed in this article are solely those of the authors and do not necessarily represent those of their affiliated organizations, or those of the publisher, the editors and the reviewers. Any product that may be evaluated in this article, or claim that may be made by its manufacturer, is not guaranteed or endorsed by the publisher.
